# A Mechanically Stimulated Co-culture in 3-Dimensional Composite Scaffolds Promotes Osteogenic and Anti-osteoclastogenic Activity and M2 Macrophage Polarization

**DOI:** 10.34133/bmr.0135

**Published:** 2025-02-05

**Authors:** Georgia-Ioanna Kontogianni, Konstantinos Loukelis, Amedeo Franco Bonatti, Elisa Batoni, Carmelo De Maria, Giovanni Vozzi, Raasti Naseem, Kenneth Dalgarno, Heungsoo Shin, Chiara Vitale-Brovarone, Maria Chatzinikolaidou

**Affiliations:** ^1^ Department of Materials Science and Engineering, University of Crete, 70013 Heraklion, Greece.; ^2^ Research Center E. Piaggio, Department of Information Engineering, University of Pisa, 56126 Pisa, Italy.; ^3^ School of Engineering, Newcastle University, Newcastle upon Tyne NE1 7RU, UK.; ^4^ Department of Bioengineering, Hanyang University, Seoul 04763, Korea.; ^5^ BK21 FOUR, Education and Research Group for Biopharmaceutical Innovation Leader, Hanyang University, Seoul 04763, Korea.; ^6^ Department of Applied Science and Technology, Politecnico di Torino, 10129 Turin, Italy.; ^7^ Foundation for Research and Technology Hellas (FORTH)-IESL, 70013 Heraklion, Greece.

## Abstract

Bone is subjected to a plethora of mechanical stresses, which have been found to directly influence the equilibrium between bone resorption and formation. Taking this into account, we present herein a novel biomimicking 3-dimensional model that applies cyclic uniaxial compression onto cells co-cultured on 3-dimensionally printed scaffolds consisting of poly L-lactic acid/poly(ε-caprolactone)/poly(3-hydroxybutyrate-*co*-3-hydroxyvalerate)/Sr–nanohydroxyapatite. The aim is to investigate how compression can modulate the balance between osteogenesis and osteoclastogenesis in co-culture, as well as the polarization of macrophages. One of the key aspects of the current study is the unprecedented development of a growth-factor-free co-culture, sustainable solely by the cross talk between human bone marrow mesenchymal stem cells and human peripheral blood mononuclear cells for their survival and osteogenic/osteoclastogenic differentiation capacity, respectively. Real-time polymerase chain reaction gene expression analysis of the mechanically stimulated constructs revealed up-regulation of the osteogenesis-related markers osteocalcin, osteoprotegerin, and runt-related transcription factor 2, with concurrent down-regulation of the osteoclastogenic markers dendritic-cell-specific transmembrane protein, nuclear factor of activated T cells 1, and tartrate acid phosphatase. The secretion of the receptor activator of nuclear factor kappa-Β ligand and macrophage colony-stimulating factor, as determined from enzyme-linked immunosorbent assay, was also found to depict lower levels compared to static conditions. Finally, macrophage polarization was examined via confocal imaging of tumor necrosis factor-α and interleukin-10 secretion levels, as well as through nitric oxide synthase and arginase 1 markers’ gene expression, with the results indicating stronger commitment toward the M2 phenotype after mechanical stimulation.

## Introduction

The field of bone tissue engineering (BTE) stands at the forefront of regenerative medicine, holding tremendous promise for the repair and regeneration of damaged or lost bone tissue [1]. This multidisciplinary approach has revolutionized the way that we address bone-related clinical challenges, offering innovative strategies that surmount the limitations of traditional techniques such as autografts and allografts [1]. In particular, 3-dimensional (3D) printing has emerged as one of the most auspicious methodologies of the last decades for the production of scaffolds for BTE with enhanced structurability [2]. Of critical importance is the choice of scaffold materials, with synthetic biodegradable polymers taking the spotlight, since they offer a range of advantages such as consistent material characteristics and controllable degradation rates [3]. Composite scaffolds of polymeric matrices with bioceramics such as nanohydroxyapatite (nHA), the main component of native bone, have been utilized in 3D printing due to their enhanced mechanical properties, biocompatibility, and osteoinductive properties [4]. Furthermore, mineral ions, including strontium (Sr^2+^), zinc (Zn^2+^), and magnesium (Mg^2+^), have been extensively investigated for biochemical stimulation of the bone healing microenvironment [4]. Strontium, for example, stimulates the proliferation of osteoblasts and the formation of new bone through the calcium-sensing receptor, thus mediating the transduction of mitogen-activated protein kinase signaling [5]. Additionally, it has been previously reported to display anti-osteoclastogenic traits, tied to the enhancement of the low-density lipoprotein receptor-related protein 6/β-catenin/osteoprotegerin (OPG) signaling pathway in osteoblasts, leading to osteoclasts’ inactivation [6].

Bone is a highly dynamic and constantly adapting tissue type, undergoing a perpetual cycle of remodeling throughout an individual’s life. This intricate process, orchestrated by a balance between the antagonistic activity of bone-forming osteoblasts and bone-resorbing osteoclasts, ensures the maintenance of bone integrity, adaptation to mechanical demands, and repair of microlesions [7]. Despite their importance in the dynamic behaviors of bone tissue, the evaluation of cell responses in BTE constructs is commonly performed in static culture conditions, completely omitting the dynamic mechanical cues’ interplay with the different bone cell types and their effect on their biochemical activities [7]. As a result, recent research studies have shifted their interest toward dynamic culture conditions [8], in an attempt to include various mechanical stimuli during the development of potential BTE scaffolds. Studies have shown that different mechanical stimulation techniques can impactfully enhance the osteogenic differentiation potential of precursor cells, compared to that in static conditions [9]. Interestingly, tensile stretch, 3-point bending, and fluid flow performed in monoculture systems have proved to alter the osteoblastic cell lines’ receptor activator of nuclear factor kappa-Β ligand (RANKL)/OPG secretion ratio, favoring the production of OPG, which in turn binds to RANKL molecules and therefore obstructs osteoclastogenesis [10]. Continuous compression loading for 48 h in a co-culture model of osteoblasts and osteoclasts resulted in up-regulated osteoblasts’ activity and down-regulation of osteoclastogenesis, based on the aforementioned mechanism of RANKL/OPG interaction [11]. On a similar note, subjection of cultures to other dynamic conditions such as the application of hydrostatic pressure [12] also revealed negative regulation of osteoclastogenic potential, as determined by reduced levels of tartrate-resistant acid phosphatase (TRAP) enzyme activity. In another study, short-term mechanical stress strongly inhibited osteoclastogenesis through decreased protein expression levels of dendritic-cell-specific transmembrane protein (DC-STAMP), an important protein for osteoclasts’ formation [13].

During bone remodeling, immunomodulation governs one of the most important processes of physiological healing. Unexpected inflammation caused by implant transplantation is one of the most common reasons of BTE constructs’ failure in clinical applications, which can elicit a series of adverse reactions, among them being the rejection of the implant [14]. Macrophages, being the main immunomodulatory regulators, are able to adopt 2 chief polarization states, the activated inflammatory M1 phenotype and the anti-inflammatory and wound healing M2 phenotype [15]. M1-activated macrophages express high levels of inducible nitric oxide synthase (iNOs) to metabolize arginine and produce nitric oxide (NO), interleukin-12, and tumor necrosis factor-α (TNF-α) [15]. M2 macrophages release anti-inflammatory mediators, such as interleukin-10 (IL-10) and arginase 1 (Arg1), an enzyme that converts arginine to ornithine for the synthesis of polyamines and collagen [15]. Piezo1, a nonselective Ca^2+^-permeable cation channel, has been recently demonstrated to promote the secretion of a plethora of immunoregulation-related biomolecules, under the application of mechanical stimulation [16]. Additionally, the application of mechanical stretching enhanced macrophages’ M2 commitment and suture stem cells’ osteogenic differentiation [17]. However, the exact mechanisms behind macrophage polarization and its connection with bone regeneration are not yet fully unraveled.

Until recently, scaffolds’ evaluation in vitro often relied on monocultures, where individual cell types like osteoblasts and osteoclasts or their precursors were used [18]. While informative, monocultures inherently lack the complexity of the bone microenvironment and the cross-talk communication naturally occurring between osteoblasts and osteoclasts, limiting their ability to provide a comprehensive understanding of scaffold performance [19]. Thus, co-culture systems have been introduced as a veritable methodology to overcome these limitations. In particular, co-cultures of osteoblasts and osteoclasts have gained prominence, allowing for the study of bone-forming and bone-resorbing cells and their interaction with bone engineering constructs, thus providing a more holistic overview of bone-microenvironment-related phenomena [20]. Co-culture systems, in their majority, are performed with the addition of stimulating agents for the induction of precursor cells to osteoblasts (l-ascorbic, dexamethasone, and β-glycerophosphate) and osteoclasts (macrophage colony-stimulating factor [M-CSF] and RANKL) [20]. The addition of those biochemical molecules affects cells’ differentiation, since l-ascorbic, dexamethasone, and β-glycerophosphate promote osteoclastogenesis [21,22], while M-CSF and RANKL are naturally produced by mature osteoblasts [23]. Consequently, the exogenous supplementation of co-culture systems with these factors can overshadow physiological cross-talk occurrence monitoring, providing limited insight on how native intracellular communication takes place.

In a previous study, we examined the effect of cyclic uniaxial compression on preosteoblastic cells seeded on polymeric scaffolds [24]. In a recent report, we established a growth-factor-free co-culture system of human bone marrow mesenchymal stem cells (hBM-MSCs) and human peripheral blood mononuclear cells (hPBMCs) [25] and showed that strontium–nanohydroxyapatite (Sr–nHA)-enriched composite scaffolds favor osteogenesis and suppress osteoclastogenesis in mono- and co-cultures. Capitalizing on these findings, in the current study, we delve deeper into the effect of cyclic uniaxial compression on (a) the responses of hBM-MSCs and hPBMCs within composite scaffolds consisting of poly L-lactic acid (PLLA)/poly(ε-caprolactone) (PCL)/poly(3-hydroxybutyrate-co-3-hydroxyvalerate) (PHBV) polymeric blend enriched with 2.5% Sr–nHA, in a supplement-free co-culture, and (b) the immunomodulation of macrophages toward M1/M2 phenotypes as a prediction of the scaffolds’ fate following implantation. Cyclic uniaxial compression was applied daily. Monocultures of each cell type were cultured under the same conditions as the reference. Static groups without mechanical loading were used as control. Cell morphology was examined via scanning electron and confocal laser microscopy (CLSM). The differentiation potential of cells was determined through alkaline phosphatase (ALP) and TRAP activity biochemical assays. The gene expression of osteogenic osteonectin (OSN), osteocalcin (OSC), OPG, and runt-related transcription factor 2 (RUNX2) and osteoclastogenic DC-STAMP, nuclear factor of activated T cells 1 (NFATc1), and TRAP markers was evaluated by means of quantitative real-time polymerase chain reaction (RT-PCR), while RANKL and M-CSF expressions were determined by enzyme-linked immunosorbent assay (ELISA). Moreover, macrophages’ morphology and TNF-α and IL-10 expression were investigated through CLSM and gene expression of iNOs2 and Arg1.

## Materials and Methods

### Scaffolds’ synthesis and sterilization

**Fig. 1.**
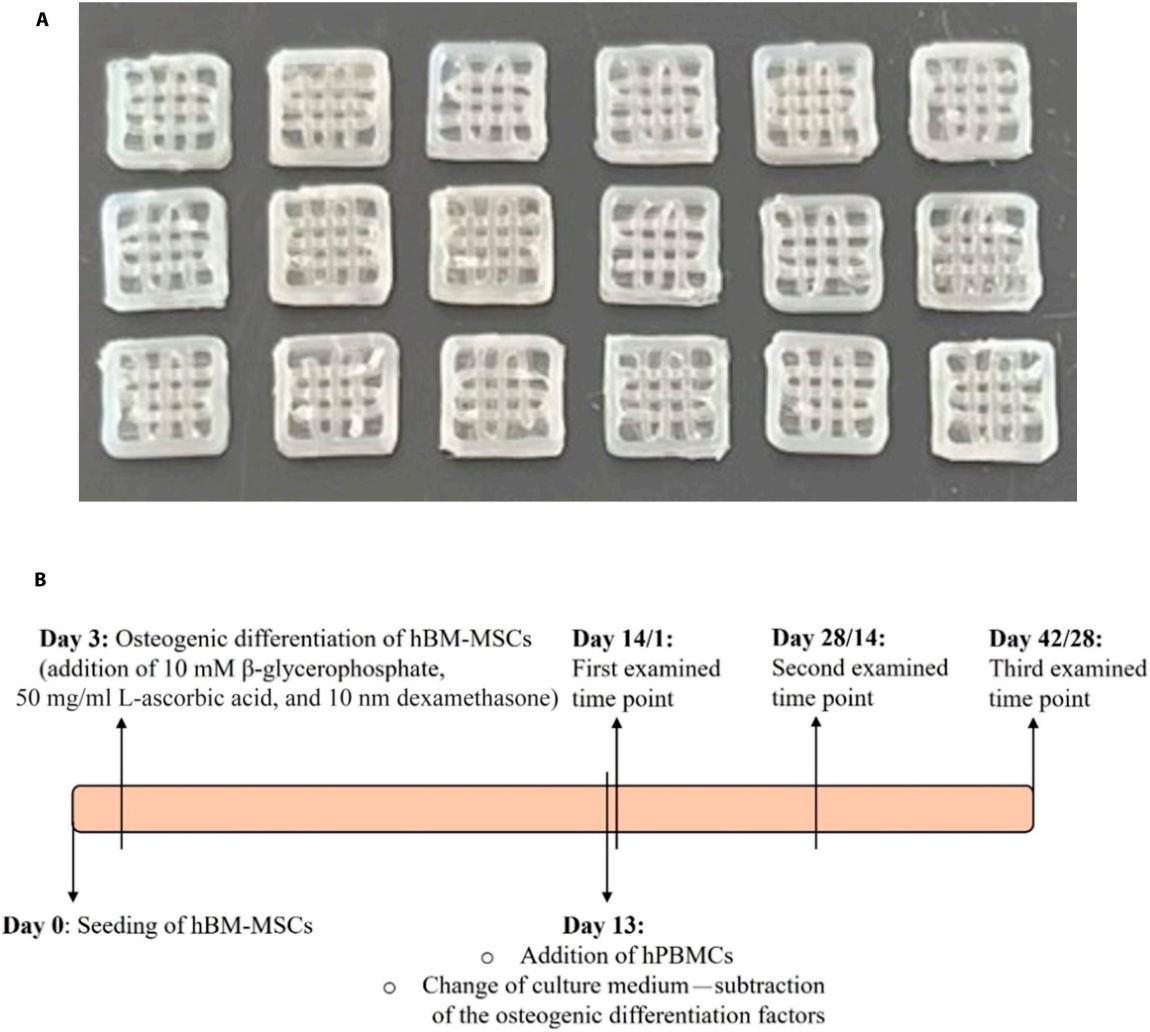
Macroscale images of the 3-dimensionally printed scaffolds (A) and schematic representation of the co-culture experimental setup for a total duration of 42 d (B). hBM-MSCs, human bone marrow mesenchymal stem cells; hPBMCs, human peripheral blood mononuclear cells.

Filaments of a 3-component PLLA/PCL/PHBV polymer blend (designated as blend) at a ratio of 90/5/5 wt.%, enriched with 2.5% Sr–nHA paste (50% Sr^2+^ substitution into nHA), were prepared using extrusion (Rondol Microlab 10-mm twin-screw extruder, as previously described [26]) and enriched with 2.5% Sr–nHA paste (50% Sr^2+^ substitution into nHA) [27] for the creation of a polymer composite filament. Cubic scaffolds of 5-mm side and 1-mm height (Fig. 1A) were produced for the biological experiments with a custom-made fused deposition modeling printer [27]. The cubic geometry was chosen based on finite element modeling analysis with a default of 50% porosity of all layers, except from the bottom layer that was printed without pores (closed) to ensure the maintenance of the cells during cell seeding and culture [24]. Prior to cell seeding, the composite scaffolds were sterilized in a 2-step procedure, by immersion in 70% ethanol for 3 min and by ultraviolet irradiation for 30 min.

### Co-culture of hBM-MSCs and hPBMCs

#### hBM-MSCs’ isolation

Bone marrow samples were collected from healthy adult donors undergoing an orthopedic surgery [25], after informed consent was provided and institutional ethics approval was granted (license number: 26-05-2010/3910, University Hospital, Heraklion, Greece). A density gradient medium (Lymphoprep, Axis-Shield, Oslo, Norway) was added though the central hole of SepMate, and addition of the sample, diluted 1:1 in phosphate-buffered saline (PBS), followed. The samples were centrifuged at 1,200*g* for 20 min at room temperature, and the hBM-MSC fraction was collected and washed twice with PBS. The isolated and purified fraction was suspended in complete alpha minimum essential medium (a-MEM) supplemented with 10% fetal bovine serum (FBS) (PAN-Biotech, Germany), 2 mM l-glutamine (Gibco, USA), 100 μg/ml penicillin/streptomycin (pen/strep; Gibco, USA), and 2.5 μg/ml fungizone (amphotericin B) (Gibco, USA) and cultured in a humidified incubator at 37 °C and 5% CO_2_ (Heal Force, China).

#### hPBMCs’ isolation

hPBMCs were isolated with the exact same protocol with hBM-MSCs, suspended in 15% FBS (PAN-Biotech, Germany), 2 mM l-glutamine (Gibco, USA), 100 μg/ml pen/strep (Gibco, USA), and 2.5 μg/ml fungizone (amphotericin B) (Gibco, USA) and frozen at −80 °C until use.

#### Immunophenotypic characterization of hBM-MSCs and hPBMCs

Trypsinized hBM-MSCs from P2 were immunophenotypically characterized using anti-CD73 (AD2; Becton Dickinson-Pharmingen, San Diego, CA, USA), anti-CD90 (F15.42; Immunotech/Coulter), anti-CD105 (SN6; Caltag, Burlingame, CA, USA), anti-CD45 (IMMU19.2; Immunotech/Coulter), anti-CD14 (RMO52; Immunotech/Coulter), and anti-CD34 (QBend10; Beckman-Coulter) monoclonal antibodies. Data were processed in Beckman Coulter Cytomics FC 500 (Coulter, Miami, FL, USA) and had been presented previously [25].

hPBMCs were stained with the antibodies CD45–PC7 and CD14–phycoerythrin (both from Beckman Coulter). Stained samples were then analyzed for CD45+ and CD14+ cells. Analysis was performed in a Cytomics FC 500 flow cytometer (Beckman Coulter, Brea, CA, USA) [25].

#### Co-culture protocol

The cell seeding procedure was performed as previously described [25]. Briefly, the composite scaffolds, after the sterilization process, were immersed in complete a-MEM containing 100 μM l-ascorbic acid 2-phosphate (Sigma, Darmstadt, Germany) for 45 min. The culture medium was removed, and the scaffolds were left for air-drying for 1 h. Subsequently, hBM-MSCs were seeded on top of the scaffolds at a cell density of 7 × 10^4^ cells/scaffold and left for 3 d in complete a-MEM supplemented with 100 μM l-ascorbic acid 2-phosphate, in order to adhere. After the 3 d of culture, osteogenic differentiation of hBM-MSCs started with the addition in the culture medium of 10 nM dexamethasone (Sigma, Darmstadt, Germany), 10 mM β-glycerophosphate (Sigma, Darmstadt, Germany), and 50 μg/ml l-ascorbic acid 2-phosphate for 10 d. hPBMCs were added after 13 d of hBM-MSC culture at a cell density of 5 × 10^5^ cells/scaffold, and the culture medium was changed to a-MEM supplemented with 7.5% FBS, 7.5% heat-inactivated human serum, 2 mM l-glutamine, 100 μg/ml pen/strep, and 2.5 μg/ml fungizone. Monocultures of both cell types were cultured under the same culture conditions as control cultures, while for the hPBMC monoculture, 25 ng/ml M-CSF (PreproTech, France) and 50 ng/ml RANKL (PreproTech, France) were added to the culture medium, to guide multinucleation and osteoclast formation, in order to ensure that the isolated hPBMCs have the capacity to perform multinucleation and form osteoclasts and for the comparison of the co-culture results with those of the reference monoculture experiment. The co-culture was performed for 28 d without the addition of any supplementary growth factors, with medium changes twice weekly, and 3 distinct time points were chosen to evaluate the co-culture. The designation of the mono- and co-culture time points includes 2 numbers for the days, 14/1, 28/14, and 42/28. The first number relates to the time of hBM-MSCs in culture, and the second number indicates the time of hPBMCs in culture. The co-culture experimental setup is presented in Fig. 1B.

#### Uniaxial cyclic compression of co-culture

Uniaxial cyclic compression was applied on the cell-seeded scaffolds using an MCTX bioreactor (CellScale, Waterloo, Canada), as previously described [24]. Briefly, the cell-seeded surface of the composite scaffolds was turned at 90° for mechanical stimulation at 1 Hz-frequency, with the strain equal to 8% of the scaffold side (400 μm of displacement) for 30 min/d for the total duration of the co-culture. The application of mechanical stresses started 3 d after the cell seeding of hBM-MSCs, i.e., after the addition of the osteogenic cocktail in the cell culture. Mechanical stimulation was performed daily until the addition of the hPBMCs, after which the culture was left to rest for 4 d. After this time point, stimulation was applied to the co-culture every other day. Control static cultures were maintained under identical culture conditions, without the application of uniaxial cyclic compression, to monitor the impact of mechanical stimulation on the cell viability and differentiation of the cells.

#### Cell adhesion and morphology

The cell adhesion and morphology of hBM-MSCs and hPBMCs was monitored with scanning electron microscopy (SEM). Briefly, cells were washed twice with PBS, at 14/1 and 28/14 d in culture, and fixed with 4% *v*/*v* paraformaldehyde (PFA) for 20 min at room temperature. Samples were dehydrated in increasing concentrations of ethanol (30% *v*/*v* to 100% *v*/*v*). Cell-seeded scaffolds were then dried in a critical point drier (Baltec CPD 030, Baltec, Los Angeles, CA, USA), sputter-coated with a 20-nm-thick layer of gold (Baltec SCD 050, Baltec, Los Angeles, CA, USA) and observed under a microscope at an accelerating voltage of 20 kV.

CLSM was used to visualize the adhesion and proliferation of hBM-MSCs and hPBMCs. Briefly, cells, at the corresponding time points, were washed with PBS and fixed with 4% PFA for 20 min. Cell permeabilization and blocking were performed with the addition of 0.1% bovine serum albumin (BSA) in 0.1% Triton X-100 for 30 min. Cytoskeletal actin was stained with tetramethyl-rhodamine B isothiocyanate-conjugated phalloidin (TRITC-phalloidin conjugate, Sigma-Aldrich, St. Louis, MO, USA) for 1 h according to the manufacturer’s instructions, and cell nuclei were stained with 4′,6′-diamidino-2-phenylindole dihydrochloride (DAPI; Invitrogen, Thermo Fisher Scientific, Waltham, MA, USA) for 5 min. The samples were washed with PBS and observed under a confocal microscope (TCS SP8, Leica).

#### Biochemical determination of cell differentiation

##### ALP activity

ALP activity was determined after 14/1, 28/14, and 42/28 d in culture in mono- and co-cultures of hBM-MSCs under static and dynamic conditions, based on a protocol that had been previously described [27]. Briefly, the cells at each time point were rinsed with PBS and lysed with 100 μl of lysis buffer (0.1% Triton X-100 in distilled water) and subjected to 2 freeze–thaw cycles. An aliquot of 50 μl was mixed with 50 μl of ALP reaction solution (2 mg/ml *p*-nitrophenyl phosphate [Sigma, USA] in 50 mM Tris-HCl and 2 mM MgCl_2_ at pH 10.5), and the samples were incubated at 37 °C for 1 h. The absorbance of the reaction was measured at 405 nm in a spectrophotometer (Synergy HTX Multi-Mode Microplate Reader, BioTek, USA) and translated to *p*-nitrophenol (pNP) concentrations with the use of a calibration curve, constructed from known pNP values. The activity of the enzyme was expressed as nanomoles of pNP/minute and normalized cell number, previously determined with PrestoBlue assay.

##### TRAP activity

TRAP activity was determined at 28/14 and 42/28 d of culture in mono- and co-cultures of hPBMCs under static and dynamic conditions, based on a previously described protocol [25]. Briefly, the cells at each time point were rinsed with PBS and lysed with 100 μl of lysis buffer (0.1% Triton X-100 in distilled water) and subjected to 2 freeze–thaw cycles. An aliquot of 50 μl was mixed with 50 μl of TRAP reaction solution (2 mg/ml *p*-nitrophenyl phosphate in 0.1 M glycine with 20 mM sodium tartrate and 2 mM MgCl_2_ at pH 5), and the samples were incubated at 37 °C for 1 h. The absorbance of the reaction was measured at 405 nm in a spectrophotometer (Synergy HTX Multi-Mode Microplate Reader, BioTek, USA) and translated to pNP concentrations with the use of a calibration curve, constructed from known pNP values. The activity of the enzyme was expressed as nanomoles of pNP/minute and normalized to total cellular protein in cell lysates determined using Bradford assay (AppliChem, Germany).

#### ALP expression and visualization by means of CLSM

ALP staining was chosen to evaluate the differentiation of the hBM-MSCs on the composite scaffold under static and dynamic conditions with CLSM, in mono- and co-cultures, according to the manufacturer’s instructions after 28/14 d in culture [28]. Briefly, the cells were washed twice with a-MEM (basal, without the addition of supplements) and stained with ALP staining (Thermo Fisher Scientific, Waltham, MA, USA), diluted 1/500 in basal medium. Cell nuclei were stained with Hoechst stain (Thermo Fisher Scientific, Waltham, MA, USA). Samples were washed with basal medium and observed under a confocal microscope (TCS SP8, Leica) within 90 min after staining.

#### TRAP expression and visualization by means of optical microscopy

hPBMC multinucleation and therefore osteoclastic formation were assessed microscopically though TRAP staining, in mono- and co-cultures under static and dynamic conditions after 42/28 d of the culture, according to the manufacturer’s instructions [29]. Briefly, the cells were washed 3 times with PBS and fixed with 4% PFA for 20 min. Cells were washed again with PBS and stained with TRAP solution containing 0.07 mg/ml fast garnet base solution, 0.01 M sodium nitrite solution, 0.125 mg/ml naphthol AS-BI phosphoric acid solution, 0.025 M acetate solution, and 0.00335 M tartrate solution. Cells were observed using an inverted light microscope under a ×20 lens (Axiovert 200, Carl Zeiss, Berlin, Germany), and photographs were taken by employing a ProgRes CF scan camera and its compatible software ProgRes Capture Pro (Jenoptik Optical Systems GmbH, Berlin, Germany).

#### RT-PCR for gene expression

Total RNA extraction was performed after 14/1, 28/14, and 42/28 d of culture using TRIzol reagent (Invitrogen Life Technologies, USA) according to the manufacturer’s instructions. The total RNA concentration and purity were determined using Nanodrop ND 1000 (Thermo Fisher Scientific, Waltham, MA, USA). Complementary DNA synthesis was carried out using PrimeScript RT Reagent Kit (Perfect Real Time) (TAKARA, Japan) according to the manufacturer’s instructions. The KAPA SYBR Fast Master Mix (2x) Universal (Kapa Biosystems) was used for RT-PCRs that were performed in a CFX Connect Bio-Rad RT-PCR system (Bio-Rad, USA) on markers of OSC, OSN, OPG, and RUNX2 for osteogenesis and DC-STAMP, NFATc1, and TRAP for osteoclastogenesis. The Primer-Blast software (http://www.ncbi.nlm.nih.gov/BLAST) was used for the primer design (Table 1). Amplification profiles for PCR were optimized for primer sets. For OSN, OPG, RUNX2, DC-STAMP, NFATc1, and TRAP, RT-PCR was run at 95 °C for 3 min, followed by 40 amplification cycles at 95 °C for 3 s and 58 °C for 30 s. For OSC, the reaction was run at 95 °C for 3 min, followed by 40 amplification cycles at 95 °C for 3 s and 56 °C for 30 s. The run was completed with the dissociation curve beginning at 65 °C for 5 s and increasing to 95 °C with 0.5 °C increments. Data were analyzed with Bio-Rad CFX Manager software version 3.0. Relative expression of target genes was calculated using the ΔΔCq (where Cq is the threshold cycle) after normalization to 2 housekeeping genes evaluated by BestKeeper (beta-2-microglobulin and succinate dehydrogenase complex, subunit A). Triplicate samples for each time point were analyzed (*n* = 3).

**Table 1.** Primers designed for the real-time PCR analysis of several osteogenic and osteoclastogenic differentiation-related genes and the respective amplicon size of the PCR products for BM-MSCs

Gene symbol	Forward (5′–3′)	Reverse (5′–3′)	Amplicon size (bp)
B2M	TGTCTTTCAGCAAGGACTGGT	ACATGTCTCGATCCCACTTAAC	138
SDHA	GCATGCCAGGGAAGACTACA	GCCAACGTCCACATAGGACA	127
OPG	GTGTGCGAATGCAAGGAAGG	AGCAGGAGACCAAAGACACTG	209
OSC	GTGCAGCCTTTGTGTCCAAG	TCAGCCAACTCGTCACAGTC	157
OSN	GAAACCGAAGAGGAGGTGGTG	AGAAGTGGCAGGAAGAGTCGAA	196
RUNX2	TCTCCGCAGGTCACTACCAG	GCTGAAGAGGCTGTTTGATGC	131
DC-STAMP	CCACAGAGGTGTTGTCCTCC	CCACAAGGGCCCAAAAATCG	109
NFATc1	GTCTGGGAGATGGAAGCGAAAACT	CTGGTACTGGCTTCGCTTTCTCTT	111
TRAP	GGCAGGCAGGGAGGGAATAAA	AGTCACCCACGGCTACAAAGC	200

PCR, polymerase chain reaction; BM-MSCs, bone marrow mesenchymal stem cells; B2M, beta-2-microglobulin; SDHA, succinate dehydrogenase complex, subunit A; OPG, osteoprotegerin; OSC, osteocalcin; OSN, osteonectin; RUNX2, runt-related transcription factor; DC-STAMP, dendritic-cell-specific transmembrane protein; NFATc1, nuclear factor of activated T cells 1; TRAP, tartrate-resistant acid phosphatase

#### ELISA of RANKL and M-CSF in culture supernatants

Sandwich ELISA was performed to capture RANKL (ab213841, Abcam) and M-CSF (ab245714) cytokines’ secretion in cell culture supernatants of hBM-MSCs in mono- and co-cultures, under both static and dynamic conditions, after 14/1, 28/14, and 42/28 d in culture. ELISA was performed according to the standard protocol that was provided by the manufacturer for both kits. Absorbance was measured at 450 nm in a spectrophotometer (Synergy HTX Multi-Mode Microplate Reader, BioTek, USA) and translated to cytokine concentrations (pg/ml) with the use of a calibration curve, constructed from known values of each target protein.

### Immunomodulatory potential toward polarization of derived macrophages from bone marrow-2

#### Cell culture

Derived macrophages from bone marrow-2 (DMBM-2) cells (ACC-269, DSMZ Braunschweig, Germany) were used for the evaluation of the immunomodulatory potential of the composite scaffold under static and dynamic conditions. DMBM-2 cells were cultured in a-MEM medium (PAN-Biotech, Germany) supplemented with 10% (v/v) FBS, 100 μg/ml penicillin and streptomycin, 2 mM l-glutamine, and 2.5 μg/ml amphotericin in a humidified incubator at 37 °C and 5% CO_2_. Cells were harvested using trypsin–0.25% ethylenediaminetetraacetic acid (Gibco, Thermo Fisher Scientific, UK). For the mechanical stimulation protocol, scaffolds (5 mm × 5 mm × 1 mm) were submerged for 30 min in complete medium (a-MEM) to achieve equilibrium and therefore enhance the cell seeding process. After medium aspiration, 7 × 10^4^ cells were seeded onto each scaffold, and after 24 h, they were transferred to new well plates to exclude nonadhered cells on scaffolds.

#### Uniaxial cyclic compression of the DMBM-2-seeded scaffolds

Uniaxial cyclic compression was applied on the cell-seeded scaffolds using an MCTX bioreactor (CellScale, Waterloo, Canada), as previously described in uniaxial cyclic compression of co-culture. Briefly, the cell-seeded surface of the composite scaffolds was turned at 90° for mechanical stimulation at 1-Hz frequency, with the strain equal to 8% of the scaffold side (400 μm of displacement) for 30 min/d for the total duration of the culture period. The application of mechanical stresses started 2 d after the cell seeding of DMBM-2 and was performed every other day. Control static cultures were maintained under identical culture conditions, without the application of uniaxial cyclic compression, to monitor the impact of mechanical stimulation on cell viability and immunomodulatory potential of the applied forces.

#### Cell adhesion and morphology

After 7 and 14 d of culture, cells were washed with PBS twice and fixed with 4% PFA for 20 min. Cells were permeabilized and blocked for nonspecific binding upon incubation for 45 min with 1% BSA in 0.1% Triton X-100 in PBS. Incubation of the primary antibody, F4/80 (MCA497GA, Serotec, Oxford, UK), diluted in 0.1% PBS–BSA, for 45 min was followed by addition of the a-Rat immunoglobulin G–fluorescein isothiocyanate (STAR69, Serotec, Oxford, UK) secondary antibody and incubation for 30 min at room temperature. Between each step, scaffolds were washed 3 times with PBS. After the last wash, cytoskeletal actin was stained with the TRITC-phalloidin conjugate (Sigma-Aldrich, St. Louis, MO, USA) for 1 h according to the manufacturer’s instructions and cell nuclei were stained with DAPI (Invitrogen, Thermo Fisher Scientific, Waltham, MA, USA) for 5 min. The samples were washed with PBS and observed under a confocal microscope (TCS SP8, Leica).

#### Immunohistochemistry for macrophage polarization

Two specific markers were chosen to visualize and quantify the polarization of M0 macrophages to M1 or M2 with CLSM, TNF-α, and IL-10, respectively. After 7 and 14 d of culture, cells were washed with PBS twice and fixed with 4% PFA for 20 min. Cells were permeabilized and blocked for nonspecific binding upon incubation for 45 min with 1% BSA in 0.1% Triton X-100 in PBS. Incubation of the primary antibody, TNF-α (MCA1487, Serotec, Oxford, UK) or IL-10 (MCA1302G, Serotec, Oxford, UK), diluted in 0.1% PBS–BSA for 45 min, was followed by addition of the a-Rat immunoglobulin G–fluorescein isothiocyanate (STAR69, Serotec, Oxford, UK) secondary antibody and incubation for 30 min at room temperature. Between each step, scaffolds were washed 3 times with PBS. Cell nuclei were stained with DAPI (Invitrogen, Thermo Fisher Scientific, Waltham, MA, USA) for 5 min. The samples were washed with PBS and observed under a confocal microscope (TCS SP8, Leica).

#### RT-PCR for gene expression

RNA isolation was performed after 7, 14, and 21 d of total culture time and was used for complementary DNA synthesis and RT-PCR analysis, as described in the co-culture section, on Arg1 and iNOs. Amplification profiles for PCR were optimized for primer sets, and the primers’ design is presented in Table 2. For all genes, the RT-PCR was run at 95 °C for 3 min followed by 40 amplification cycles at 95 °C for 3 s and 58 °C for 30 s. The run was completed with the dissociation curve beginning at 65 °C for 5 s and increasing to 95 °C with 0.5 °C increments. The relative expression of target genes was calculated using the ΔΔCq (where Cq is the threshold cycle) method after normalization to 2 housekeeping genes evaluated by BestKeeper (hypoxanthine phosphoribosyltransferase-1 as a housekeeping gene and actin). Triplicate samples for each time point were analyzed.

**Table 2.** Primers designed for the real-time PCR analysis of macrophage polarization-related genes and the respective amplicon size of the PCR products

Gene symbol	Forward (5′–3′)	Reverse (5′–3′)	Amplicon size (bp)
Actin	TCGTGTTGGATTCTGGGGAC	ACGAAGGAATAGCCACGCTC	151
HPRT	TGGGCTTACCTCACTGCTTT	ATCGCTAATCACGACGCTGG	128
Arg1	GTGAAGAACCCACGGTCTGT	AGAAAGGACACAGGTTGCCC	245
iNOs	TGGTGAAGGGACTGAGCTGT	TGAGAACAGCACAAGGGGTTT	245

HPRT, hypoxanthine phosphoribosyltransferase; Arg1, arginase 1; iNOs, inducible nitric oxide synthase

### Statistical analysis

All experimental results are presented as mean ± SD. The statistical analysis was performed using the one-way analysis of variance Dunnett’s multicomparison test in GraphPad Prism version 8 software (GraphPad Software, San Diego, CA, USA). *P* values indicate statistically significant differences (*P < 0.05, **P < 0.01, ***P < 0.001, ****P < 0.0001, and *****P < 0.00001).

## Results

### Co-culture

#### Enhanced cell viability and distinct morphological changes in co-culture under static and dynamic culture conditions

**Fig. 2.**
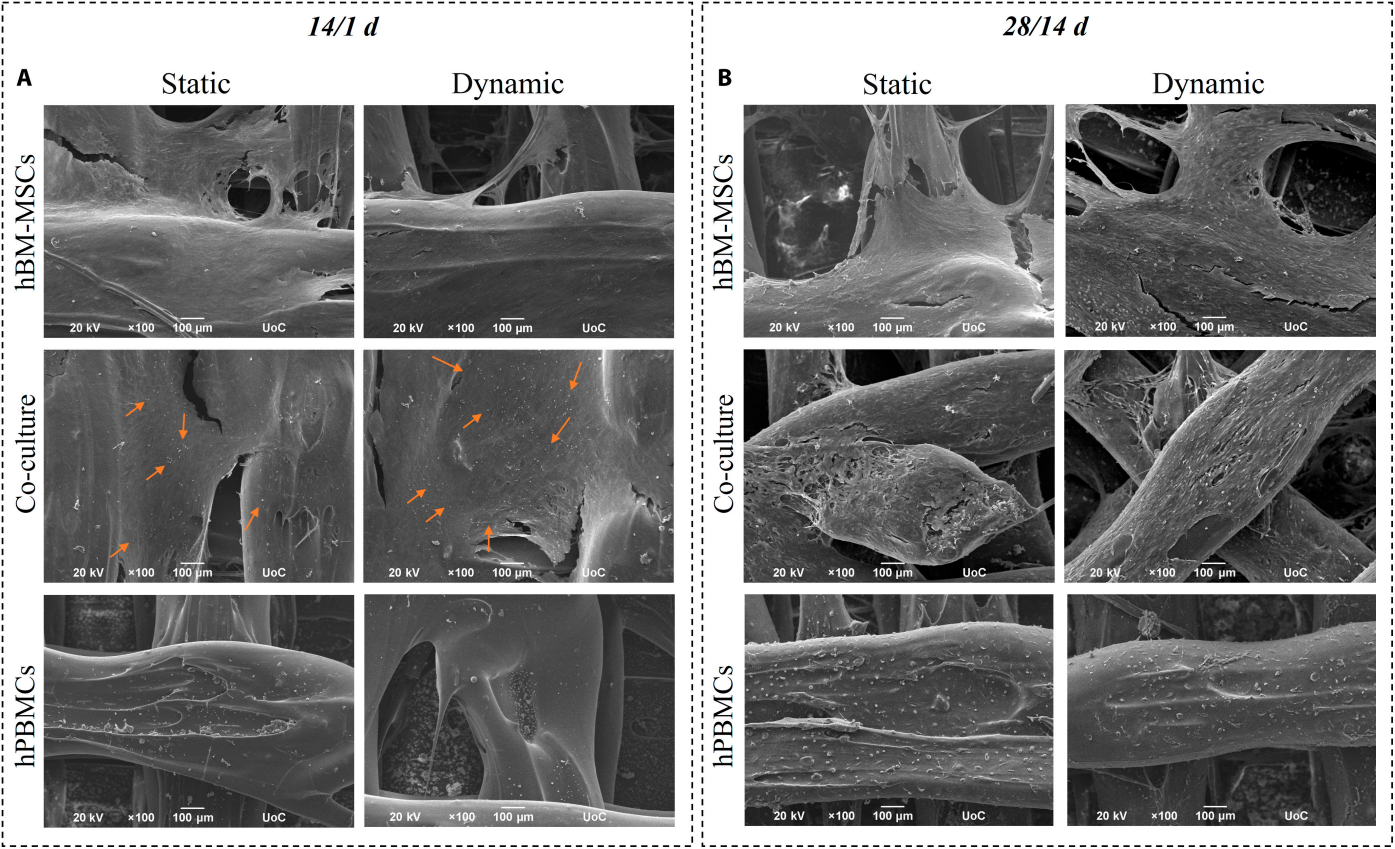
Cell morphology assessment of hBM-MSCs and hPBMCs in mono- and co-cultures. Representative scanning electron microscopy (SEM) images showing hBM-MSCs and hPBMCs in mono- and co-cultures on composite scaffolds in culture under static and dynamic conditions after 14/1 d (A) and 28/14 d (B). Original magnifications are ×100, and scale bars represent 100 μm. Orange arrows point to hPBMCs.

hBM-MSC and hPBMC morphology on 3D-printed scaffolds under the application of mechanical stimulation was evaluated through SEM imaging. After 14/1 d in culture (Fig. 2A), hBM-MSCs formed a dense cellular network on the scaffold surface under both static and dynamic cultures in mono- and co-cultures. The presence of hPBMCs was identified as round cells (pointed with orange arrows in Fig. 2A), visibly anchored on the underlying hBM-MSC layer in the co-cultures and on the surface and the bottom of the scaffolds in the monocultures, with no significant differences between static and dynamic culture conditions.

On day 28/14 of the co-culture (Fig. 2B), hBM-MSCs retained their elongated morphology with a progressively higher cell number in mono- and co-cultures, under static and dynamic conditions. Interestingly, even though hBM-MSCs had similar proliferation rates regardless of the examined condition, both dynamic mono- and co-cultures showed that hBM-MSCs had extensively undergone maturation toward osteoblasts, with a more pronounced effect on the dynamic co-culture, showcased by their rough morphology. Co-cultured hPBMCs displayed a round-shaped morphology and formed aggregates, mostly at the bottom of the scaffolds due to their innate lack of adhesion binding sites. Notably, in the hPBMC monocultures, multinucleated osteoclasts are present, as expected, due to the external application of RANKL and M-CSF, with no observable differences between the static and dynamic conditions.

**Fig. 3.**
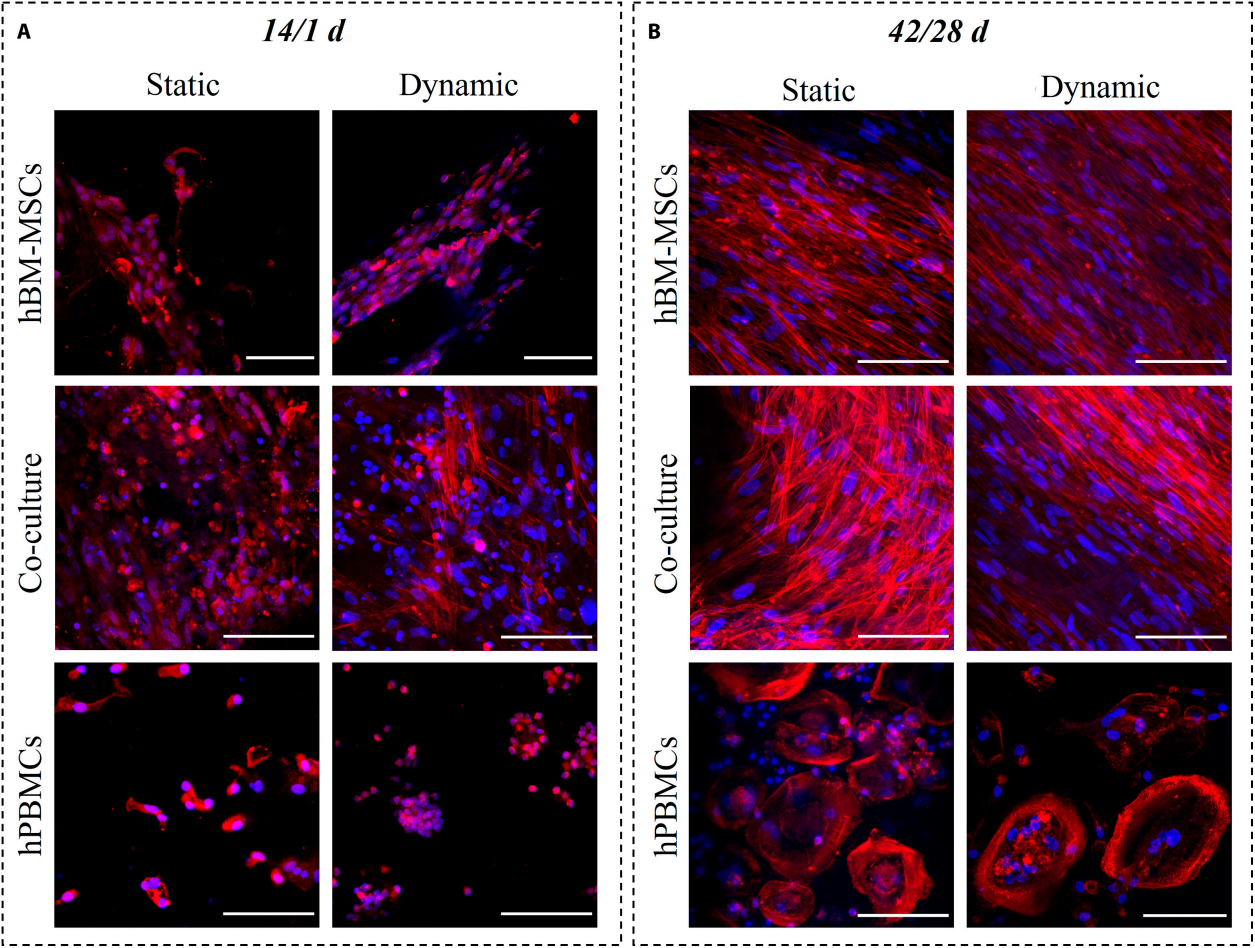
Confocal laser microscopy (CLSM) image stacks of hBM-MSCs and hPBMCs in mono- and co-cultures on composite scaffolds showing actin distribution (red) and cell nuclei (blue) after 14/1 d (A) and 42/28 d (B) in culture. Scale bars represent 100 μm.

Further investigation of cells’ morphology was also performed through CLSM and actin distribution, at 2 distinct time points of 14/1 d (Fig. 3A) and 42/28 d (Fig. 3B). More specifically, the actin distribution of hBM-MSCs in monocultures after 14/1 d, under static and dynamic conditions (Fig. 3A), revealed well-adherent cells that had formed their characteristic elongated morphology. The co-cultured hBM-MSCs showed the same morphological characteristics as the monocultured ones, with no significant differences between the static and dynamic conditions, with a few round-shaped hPBMCs captured on the hBM-MSC cell sheet. In the hPBMC monoculture, cells appear round shaped and form aggregates.

Monocultured hBM-MSCs, after 42/28 d (Fig. 3B), depicted an elongated shape with a physiological cellular network in both static and dynamic conditions, with no observable differences for the co-cultured hBM-MSCs. As aforementioned in the SEM images, hPBMCs are not present in the co-cultures after 42/28 d in culture, due to their lack of adhesion on the scaffold strands, and thus on the hBM-MSC cell sheet. Contrariwise, in the monocultures of hPBMCs, fully formed multinucleated osteoclasts are present, under static and dynamic conditions, due to exogenous supplementation with M-CSF and RANKL.

#### Increased osteogenic activity and reduced osteoclastogenic potential under dynamic culture assessed via ALP and TRAP expression through biochemical analysis and microscopy

**Fig. 4.**
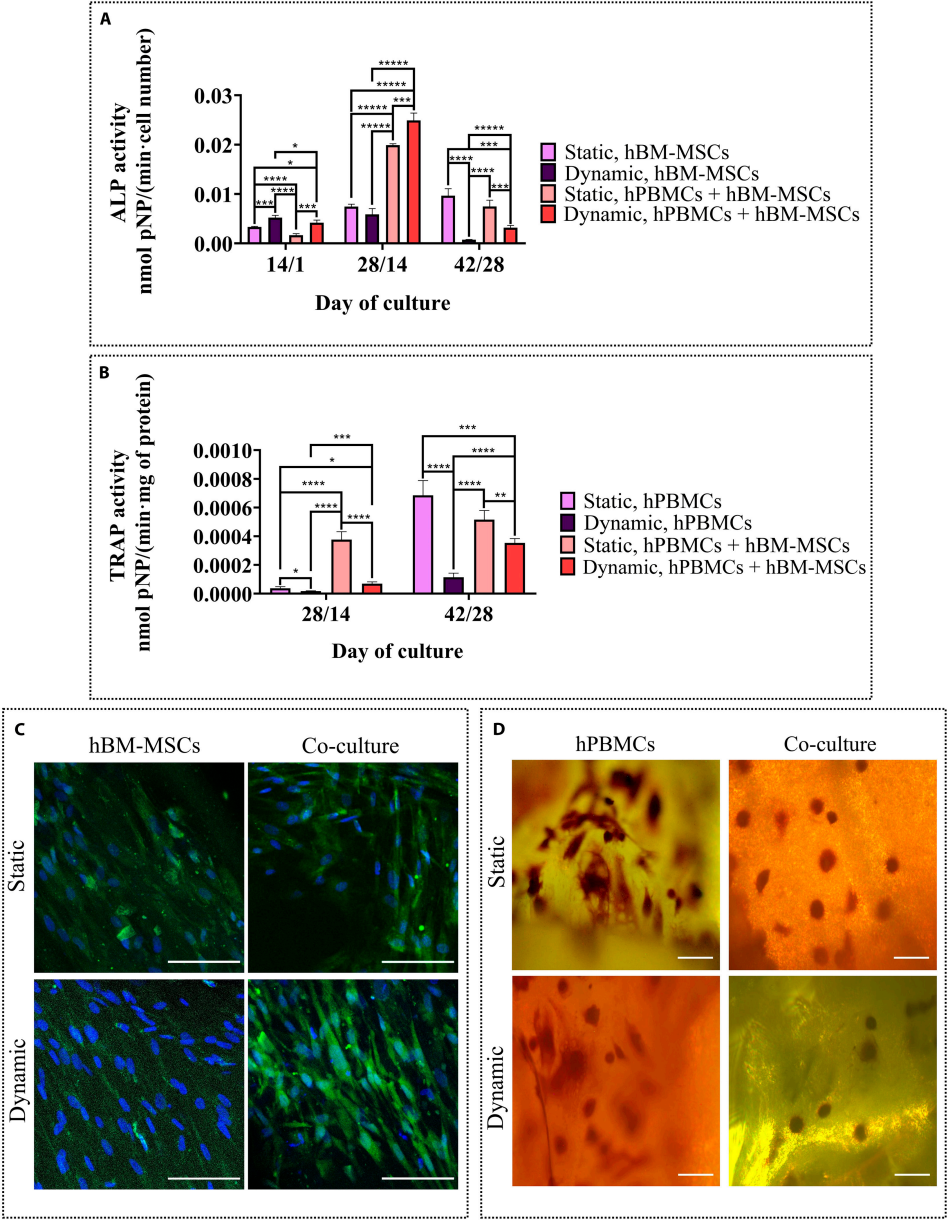
Normalized alkaline phosphatase (ALP) activity of hBM-MSCs cultured on composite scaffolds for up to 42/28 d in mono- and co-cultures (A) and normalized TRAP activity of hPBMCs (B) cultured on composite scaffolds for up to 42/28 d in mono- and co-cultures under static and dynamic conditions. Each bar represents the mean ± SD of 4 replicates (*n* = 4; *P < 0.05, **P < 0.01, ***P < 0.001, ****P < 0.0001, and *****P < 0.00001). ALP staining (green) showing hBM-MSCs on composite scaffolds in mono- and co-culture with hPBMCs for 28/14 d (C) (cell nuclei appear blue). TRAP staining showing hPBMCs in co-culture with hBM-MSCs in the absence of osteoclastogenic medium and in monoculture in the presence of osteoclastogenic medium (25 ng/ml macrophage colony-stimulating factor [M-CSF] and 50 ng/ml receptor activator of nuclear factor kappa-Β ligand [RANKL]) for 42/28 d in culture on composite scaffolds (D). Scale bars represent 100 μm. pNP, *p*-nitrophenol.

For the evaluation of the osteogenic potential of the mechanically stimulated hBM-MSCs, ALP activity was examined after 14/1, 28/14, and 42/28 d (Fig. 4A). On day 14/1, dynamically cultured hBM-MSCs in mono- and co-cultures depicted significantly higher ALP activity compared to the static controls, which depicted comparable values. Moreover, at day 28/14, co-cultured hBM-MSCs under both static and dynamic conditions revealed an almost 2-fold increase in comparison with the corresponding monocultures, and the dynamic co-culture demonstrated the highest enzyme activity among the different culture conditions. Notably, hBM-MSCs static monoculture had comparable levels of ALP activity at 28/14 and 42/28 d, while all other conditions had a dramatic decrease after 42/28 d of culture compared to the previous tested time point. These results indicated cell maturation after 28/14 d of the culture.

The osteoclastogenic potential of the hPBMC-seeded composite scaffolds was evaluated biochemically by means of TRAP activity after 28/14 and 42/28 d of culture (Fig. 4B). After 28/14 d in culture, TRAP activity is significantly up-regulated in the static co-culture compared to that in the other culture conditions. After 42/28 d, the static monoculture showed a 6-fold increase in comparison with the previous tested time point and overpassed the other tested conditions, indicating that osteoclastic differentiation is promoted when both hBM-MSCs and mechanical stimulation are not present.

ALP secretion was examined via CLSM after 28/14 d in culture (Fig. 4C), with the dynamically co-cultured hBM-MSCs having up-regulated enzyme activity compared to static co-culture and both monoculture conditions. The significantly increased ALP secretion in the dynamic co-culture is in accordance with the qualitative results of this specific time point, extracted from the biochemical determination of the enzymes’ activity (Fig. 4A). Moreover, TRAP CLSM staining (Fig. 4D) revealed single round cells in both static and dynamic co-cultures, while multinucleation was evidenced in the case of control monocultures, due to the induction media used, which contained RANKL and M-CSF.

#### Mechanical stimulation enhances osteogenic and suppresses osteoclastogenic gene expression markers in mono- and co-cultures

**Fig. 5.**
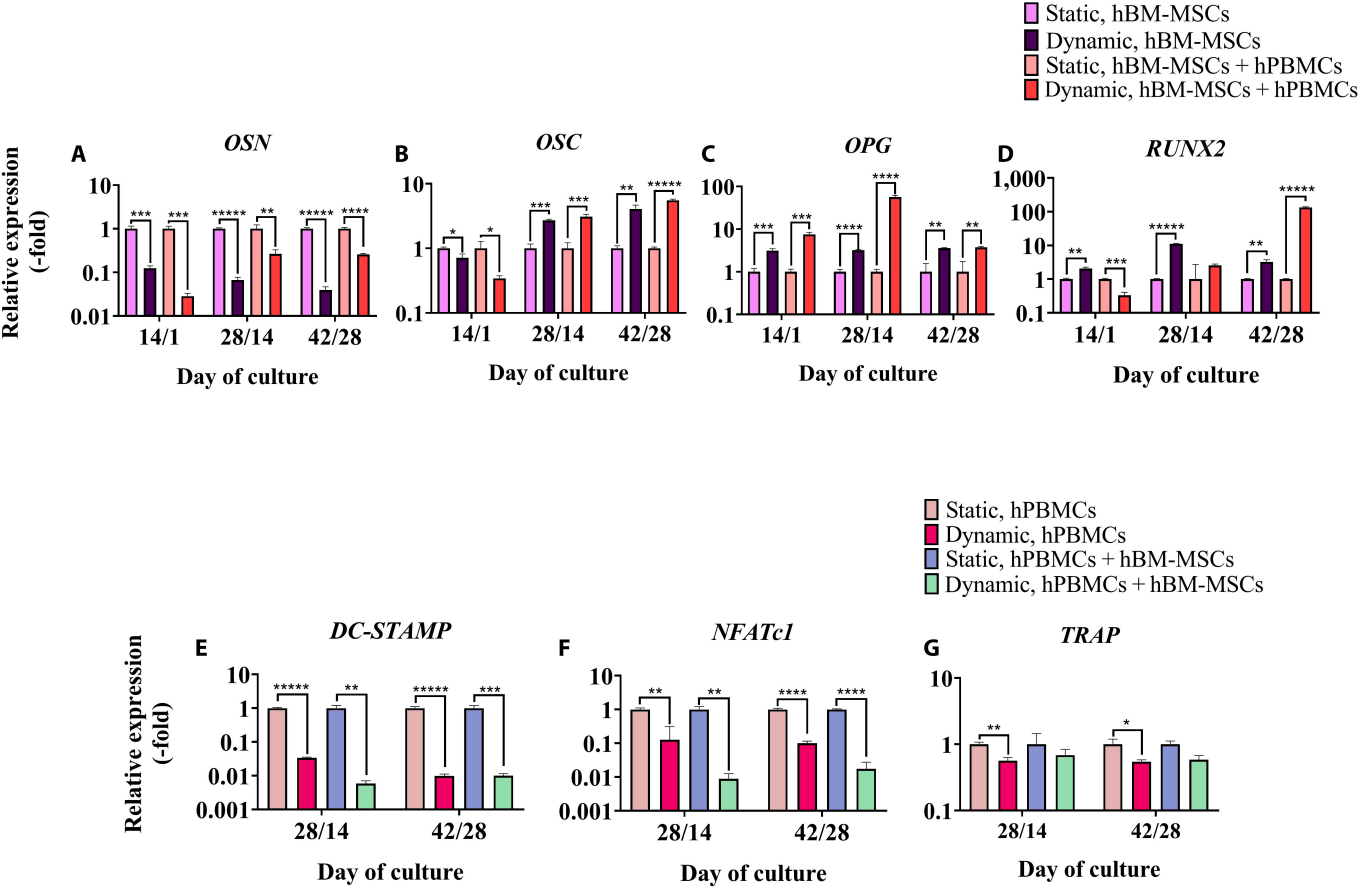
Relative osteogenic and osteoclastogenic gene expression of hBM-MSCs and hPBMCs, respectively, after 14/1, 28/14, and 42/28 d in mono- and co-cultures on composite scaffolds under static and dynamic conditions. Osteonectin (OSN) (A), osteocalcin (OSC) (B), osteoprotegerin (OPG) (C) runt-related transcription factor 2 (RUNX2) (D), dendritic-cell-specific transmembrane protein (DC-STAMP) (E), nuclear factor of activated T cells 1 (NFATc1) (F), and tartrate-resistant acid phosphatase (TRAP) (G) were analyzed. Relative gene expression levels were analyzed using the ΔΔCt method by normalizing with 2 housekeeping genes (B2M and SDHA) as endogenous controls. Each bar represents the mean ± SD of 3 replicates (*n* = 3; *P < 0.05, **P < 0.01, ***P < 0.001, ****P < 0.0001, and *****P < 0.00001).

To better comprehend the effect of mechanical stimulation on the osteogenic and osteoclastogenic properties of co-cultured hBM-MSCs and hPBMCs, the gene expression of specific markers was analyzed by means of RT-PCR after 14/1, 28/14, and 42/28 d in culture (Fig. 5). OSN (Fig. 5A), OSC (Fig. 5B), OPG (Fig. 5C), and RUNX2 (Fig. 5D) were picked as osteogenic markers and DC-STAMP (Fig. 5E), NFATc1 (Fig. 5F), and TRAP (Fig. 5G) for osteoclastogenesis. OSN was significantly up-regulated at all investigated time points in the static cultures, of both mono- and co-cultured hBM-MSCs, compared to that in dynamic culturing. However, after 28/14 and 42/28 d, OSN expression in the dynamic co-cultures showed an increased pattern. On day 14/1, OSC expression was significantly down-regulated in the dynamic cultures, while after 28/14 d, this effect was reversed. More specifically, dynamically monocultured hBM-MSCs showed a 2-fold increase compared to the static monoculture, while the dynamic co-culture showed a magnified 3-fold increase compared to static co-culture conditions at 28/14 d. The same pattern was also observed after 42/28 d, but intensified, since OSC expression showed a 4-fold increase in the hBM-MSC dynamic monoculture and a 5-fold increase in the dynamic co-culture compared to their respective static cultures. OPG expression was up-regulated at all time points in the dynamic mono- and co-cultures compared to that in the static controls. The most pronounced divergence is captured at day 28/14 of the dynamic co-culture, exhibiting a 56-fold increase compared to the static control, indicating that the presence of hPBMCs in conjunction with uniaxial compression had a synergistic effect toward osteogenesis. Notably, RUNX2 expression at the first time point of day 14/1 was found to be down-regulated in the dynamic co-culture compared to the static one. An opposite trend was evident in the monocultures, which continued up to day 42/28. After 42/28 d, RUNX2 expression in the dynamic co-culture significantly increased compared to the static counterpart by 134-fold.

Mechanical uniaxial compression led to significant suppression of hPBMC multinucleation and thus osteoclast formation. In detail, DC-STAMP (Fig. 5E) was down-regulated at both the examined time points of 28/14 and 42/28 d in hPBMC dynamic mono- and co-cultures in comparison with that in the corresponding static cultures. A similar trend was reported also for the expression of NFATc1 (Fig. 5F). In regard to the TRAP (Fig. 5G) gene marker, it was primarily down-regulated in the dynamic monocultures of hPBMCs at the middle (day 28/14) and late (day 42/28) time points compared to the static cultures, while no statistically significant differences were observed between the 2 co-culture conditions.

#### Mechanical stimulation modulates RANKL and M-CSF secretion depending on culture conditions

**Fig. 6.**
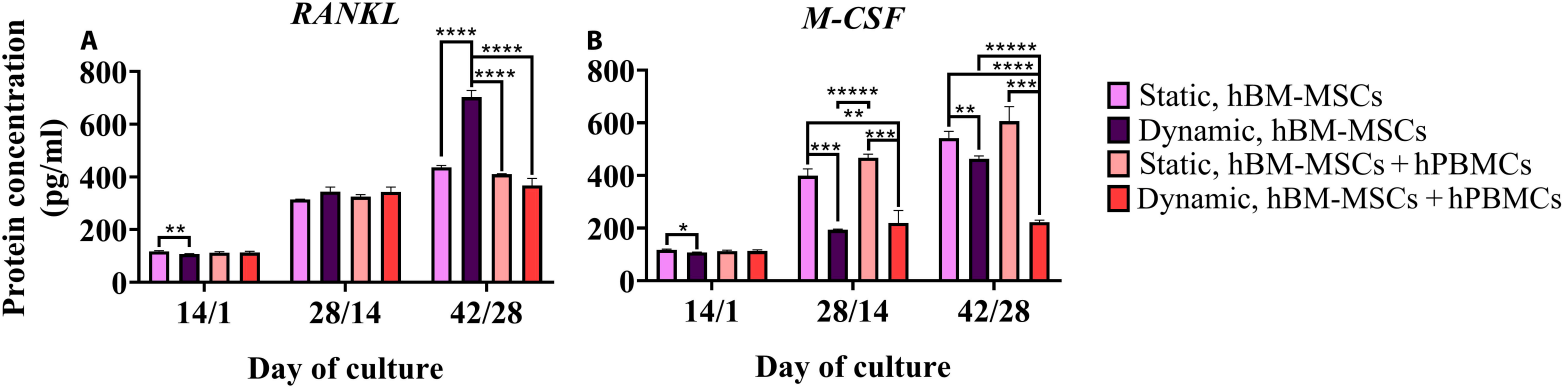
Enzyme-linked immunosorbent assay (ELISA) detection of soluble RANKL (A) and M-CSF (B) in the cell supernatants of hBM-MSCs after 14/1, 28/14, and 42/28 d in mono- and co-cultures on composite scaffolds under static and dynamic conditions.

The cytokines RANKL’s and M-CSF’s secretion levels were examined via ELISA, as they both constitute major regulators of native osteoclastogenesis. RANKL secretion (Fig. 6A) presented a statistically significant increase after 14/1 d in static monoculture compared to that in the other culture conditions. After 28/14 d, no significant differences were captured among the different types of culture, with a rise in levels for all conditions. RANKL expression after 42/28 d in culture showed comparable levels with day 28/14 for the static monoculture and both co-cultures. Interestingly, the dynamic monoculture showed a significant increase compared to the other tester conditions. M-CSF secretion (Fig. 6B) followed a similar pattern as that of RANKL after 14/1 d in culture. However, after 28/14 and 42/28 d, M-CSF secretion was significantly reduced in both dynamic cultures compared to that in the static cultures. On the 42/28 time point, the dynamic monoculture exhibited M-CSF secretion levels than the dynamic co-culture.

### Immunomodulatory potential of cell-seeded scaffolds under the influence of mechanical stimulation

#### Application of mechanical stimulation does not affect macrophage cell morphology compared to static culture conditions

**Fig. 7.**
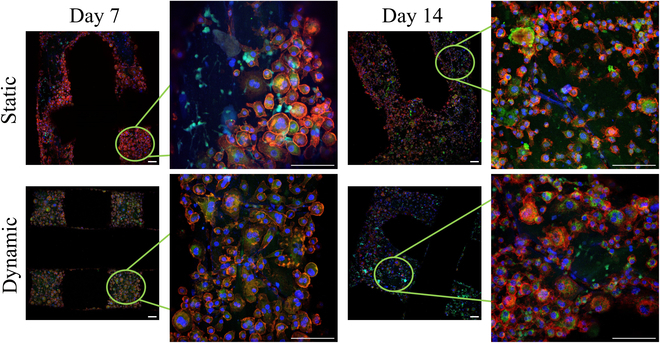
Representative CLSM image stacks of bone-marrow-derived macrophages cultures on composite scaffolds showing actin distribution (red), macrophage-specific marker F4/80 (green), and cell nuclei (blue) after 7 and 14 d in static and dynamic cultures. Scale bars represent 100 μm.

The morphology of macrophages cultured under static and dynamic conditions was observed under confocal microscopy after 7 and 14 d in culture (Fig. 7). Cells were stained against the F4/80 macrophage-specific marker (green), actin (red), and cell nuclei (blue). Mechanical stimulation did not appear to visibly affect macrophages’ characteristic round-shaped morphology and cell viability, as most cells had already formed actin networks with circular patterning from day 7.

#### Mechanical stimulation down-regulates pro-inflammatory TNF-α and up-regulates anti-inflammatory IL-10 secretion

**Fig. 8.**
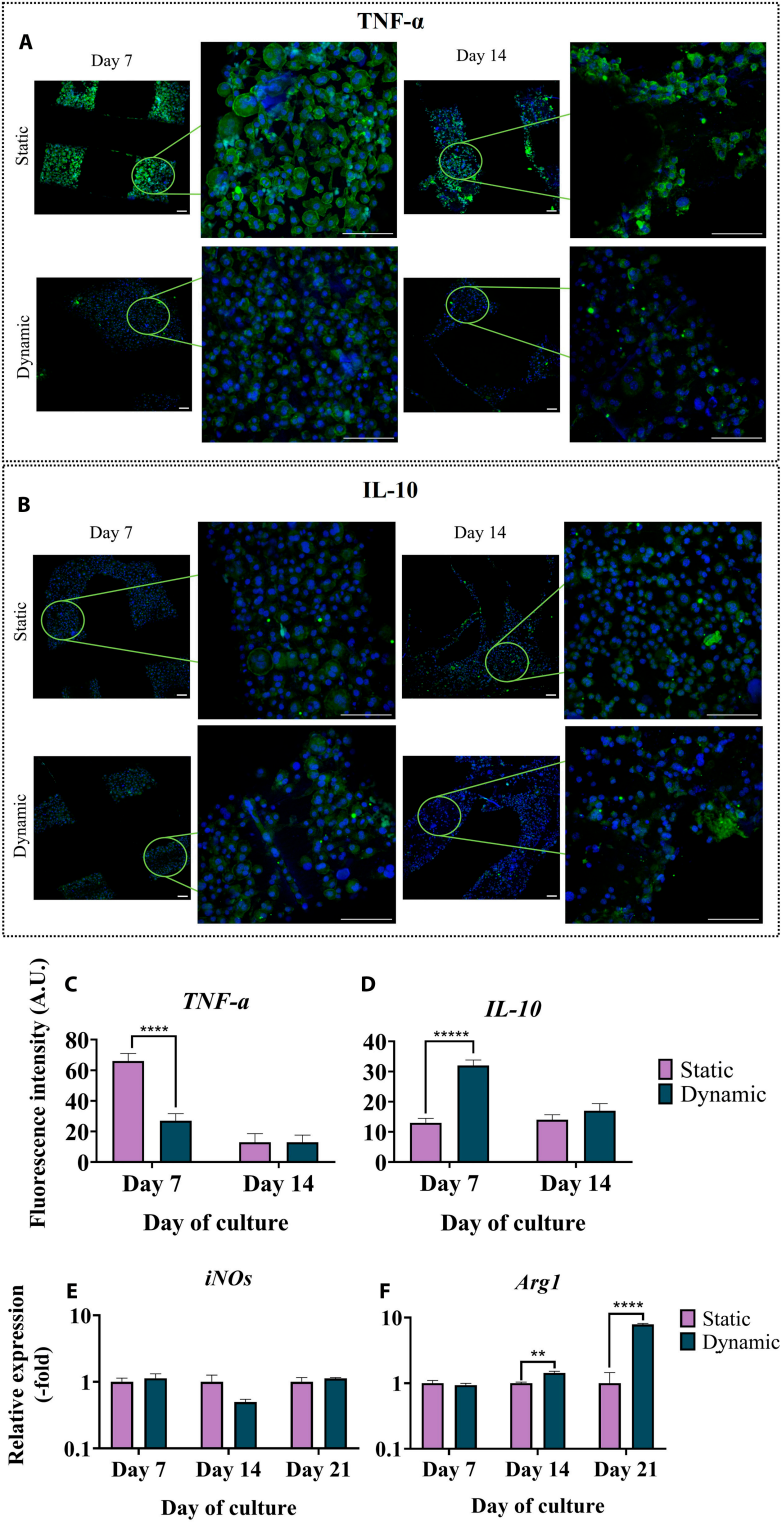
Mechanical stimulation-dependent changes in macrophage polarization markers. Representative CLSM images of the M1 polarization marker tumor necrosis factor-α (TNF-α) (green) (A) and of the M2 polarization marker interleukin-10 (IL-10) (green) (B). Quantification of the fluorescence intensity of TNF-α (C) and of IL-10 after 7 and 14 d in culture on composite scaffolds under static and dynamic conditions (D). Scale bars represent 100 μm. Each bar represents the mean ± SD of triplicates (*n* = 3; ****P < 0.0001). Relative gene expression of iNOs (M1 marker) (E) and Arg1 (M2 marker) (F) after 7 and 14 d of bone-marrow-derived macrophages in culture on composite scaffolds under static and dynamic conditions. Each bar represents the mean ± SD of triplicates (*n* = 3; **P < 0.01 and ****P < 0.0001).

For the evaluation of macrophages’ polarization tendency, TNF-α and IL-10 were used as markers for M1 and M2 phenotypes, respectively. Both markers are identified in CLSM images with a green color, while cell nuclei appear as blue. TNF-α secretion (Fig. 8A) stained after 7 and 14 d in culture under static and dynamic conditions. From the CLSM images, it is obvious that TNF-α-stained cells under static conditions after 7 and up to 14 d in culture depicted higher secretion levels, especially on day 14. The quantification results of the fluorescence intensity of TNF-α (Fig. 8C) further confirmed the qualitative imaging results, with static conditions clearly exceeding dynamic conditions on day 7, while a steep decline of comparable intensity was evidenced after 14 d for both conditions. Moreover, qualitative imaging of IL-10 secretion (Fig. 8B) after 7 d in culture showed increased cytokine intensity for the dynamic culture compared to the static culture, while after 14 d, no significant differences were captured among them. Quantitative results (Fig. 8D) verified the latter observations.

#### Dynamic culture conditions promote M2 macrophage polarization through Arg1 up-regulated gene expression

DMBM-2 commitment toward M1 and M2 phenotypes was also studied through gene expression analysis of 2 key enzymes, iNOs and Arg1, related to M1 and M2 polarization, respectively. Both the static and dynamic cultures of macrophages expressed comparable levels of iNOs (Fig. 8E) after 7, 14, and 21 d in culture, with no significant differences among the different culture conditions. In contrast, Arg1 expression (Fig. 8F) was significantly up-regulated in the dynamic culture after 14 and up to 21 d compared to the static cultures, with a 10-fold increase on day 21.

## Discussion

Mechanical stimulation is a vital physiologically occurring process, which modulates skeletal function through the regulation of bone remodeling and thus the activity of bone-forming osteoblasts and bone-resorbing osteoclasts [30]. Dynamic culture conditions have attracted researchers’ attention, since they aim to mimic all alternating mechanical forces that bone is subjected to on a day-to-day basis [30]. The complexity of the interactions between mechanical forces and cells’ behavior is a challenging system to monitor in vivo; thus, most groups have so far focused on their recapitulation under in vitro conditions [31]. Therefore, the establishment of novel dynamic culture systems is aimed toward shedding further light into the unraveling of such intricate physiologies, acting as potent biomimicking models without the mediation of costly and time-consuming in vivo experimentation.

In the context of BTE and bone graft implantation, another important aspect to take into consideration is the polarization of macrophages [15], as their ability to transit between pro-inflammatory (M1) and anti-inflammatory (M2) states dictates a significant part of the immunomodulatory response involved in de novo bone regeneration and healing processes [32]. The application of mechanical stimulation has been previously reported to affect their polarization state, favoring the M2 phenotype and thus bone tissue repair [17]. In the current study, we investigated and propose for the first time a novel growth-factor-free BTE dynamic culture approach, by applying cyclic uniaxial compression on fused deposition modeling 3D-printed Sr–nHA-substituted polymeric blend scaffolds, seeded with hBM-MSCs and hPBMCs. Macrophage cells were also seeded onto the same scaffolds, and the impact of mechanical stimulation on their phenotype commitment was investigated. Scaffolds consisted of PLLA/PCL/PHBV at a ratio of 90/5/5 wt.%, which have been optimized regarding their biomechanical properties [26]. The frequency, the strain, and the applied duration were also selected based on previous protocols on the effect of mechanical stimulation on osteogenic differentiation [24]. Additionally, the composite scaffolds were enriched with Sr–nHA, as it has been shown to induce enhanced osteogenic and anti-osteoclastogenic capabilities [25].

All of the examined conditions showed excellent cell viability, as evidenced from SEM and CLSM images. After 28/14 d in culture, hBM-MSCs appeared to have completely covered the scaffolds’ surface, in both mono- and co-cultures, while in the hPBMC monocultures, multinucleation was initiated. These findings are in accordance with reports from the literature on PHBV/PLLA [33] and PLLA/PCL matrices [34] that have shown increased biocompatibility on the blended polymers. Interestingly, even though hBM-MSCs have similar proliferation rates in all cases, in the dynamic co-culture, mature osteoblasts are present, as shown from cells’ morphology, in agreement with a previous report on co-cultured cells on chitosan–hydroxyapatite (HA) hydrogels under perfusion [35].

ALP is an enzyme that cleaves phosphate groups and deposits them in the extracellular matrix for the formation of nHA crystals. Thus, it is an important marker that is highly expressed at the early stages of osteogenesis [27]. Dynamically co-cultured hBM-MSCs reached the maximum ALP activity after 28/14 d, with significantly up-regulated enzyme activity compared to the static co-culture and the corresponding monocultures. Therefore, the application of mechanical stimulation in conjunction with the presence of hPBMCs promoted osteogenesis, as previously mentioned in a co-culture of MC3T3-E1 and RAW264.7 cells under mechanical strain [36]. TRAP is an enzyme primarily involved with the early stages of osteoclastogenesis and is an important marker of osteoclasts’ formation and regulation. TRAP activity results showed decreased levels in the dynamic cultures compared to the static cultures after 28/14 and 42/28 d. These findings provide the first indication that the application of mechanical stimulation can impart anti-osteoclastogenic properties, as previously mentioned [13]. This group applied cyclic tensile force for 24 h upon RAW264.7 cells, stimulated by RANKL, and showed decreased osteoclastogenesis in more than 40%, compared to the unstimulated control culture, throughout TRAP staining. Moreover, ALP staining showed increased enzyme secretion levels in the dynamic co-culture, while TRAP staining showed fully formed osteoclasts in the hPBMC monocultures and absence of multinucleated cells in both co-cultures, in accordance with a previously reported co-culture of cells on dentine disks under the influence of mechanical stimulation [37].

Moreover, the gene expression of the osteogenic markers OSN, OSC, RUNX2, and OPG was analyzed by means of RT-PCR. OSN is a glycoprotein, with high affinity for collagen and HA, mainly secreted at the early stages of osteogenesis [38]. The application of uniaxial compression led to significant down-regulation of OSN gene expression compared to that of the corresponding controls, indicating that osteogenic differentiation of dynamically cultured cells precedes that of the static [9]. OSC, a protein secreted by osteoblasts, aids in bone remodeling due to its abundant glutamic acid regions, which exhibit strong affinity for calcium and HA crystals, and thus plays a pivotal role in bone mineralization and calcium homeostasis [39]. OSC gene expression showcased an up-regulation at 28/14 and 42/28 d, in dynamic mono- and co-cultures, with a 10-fold increase at the latter, with these data being aligned with a previous study on OSC protein levels after the application of cyclic compression on hBM-MSCs for 2 and 3 weeks [40]. RUNX2 is a transcription factor regulating protein in bone remodeling, governing osteoblasts’ differentiation, coordinating matrix maturation and mineralization, promoting osteoblasts’ survival, and interacting with key signaling pathways to modulate bone formation and skeletal integrity [41]. In our study, RUNX2 was up-regulated after 28/14 and 42/28 d in favor of dynamic monocultures, and in the dynamic co-culture, with a 1,000-fold increase at the 42/28 d, compared to the control. This observation is corroborated by Kang et al. [9], who applied low-magnitude strain on human adipose-derived stem cells seeded on poly(caprolactone)/poly(lactic-*co*-glycolic acid)/tricalcium phosphate scaffolds and demonstrated that the stimulated group displayed a significantly increased number of RUNX2-positive cells after 7 d in culture, compared to the control; OPG acts as a decoy receptor for RANKL, thereby preventing its interaction with RANK, a receptor on the surface of osteoclast precursors, and thus inhibits osteoclast differentiation and activation to regulate bone resorption [42]. In the current study, OPG gene expression was up-regulated in the dynamically cultured hBM-MSCs in mono- and co-cultures, indicating that mechanical stimulation modulates bone resorption through up-regulated OPG expression, facilitating bone regeneration via cytokine-mediated blockade of osteoclastogenesis. Notably, the peak OPG gene expression was observed at day 28/14 in dynamically co-cultured hBM-MSCs, demonstrating a 100-fold up-regulation compared to controls, consistent with prior studies reporting enhanced OPG protein production following application of uniaxial and homogeneous mechanical tension in co-culture models [30],

Osteoclastogenesis was assessed via the expression of the genes DC-STAMP, NFATc1, and TRAP. DC-STAMP is a key regulator of cell–cell fusion during multinucleation of mononuclear cells, in order to form osteoclasts [43], and NFATc1 serves as a master transcription factor in osteoclastogenesis, driving the differentiation of osteoclast precursors and promoting their fusion to form mature osteoclasts capable of bone resorption [44]. Intriguingly, our findings revealed suppressed gene expression of both DC-STAMP and NFATc1 in dynamic mono- and co-cultures of hPBMCs, suggesting that mechanical stimulation may attenuate or completely inhibit osteoclastogenesis [13]. Additionally, TRAP is an enzyme predominantly expressed by osteoclasts and plays a crucial role in bone resorption by facilitating the breakdown of mineralized bone matrix [45]. Its gene expression was down-regulated in dynamic cultures, an effect likely mediated by the regulatory influence of NFATc1[44].

To further elucidate the interplay between osteoblasts and osteoclasts, ELISA was conducted in cell culture supernatants of hBM-MSCs to quantify soluble RANKL and M-CSF, pivotal osteoblast-secreted molecules crucial for mononuclear fusion and subsequent osteoclast formation [46]. Specifically, RANKL, a vital cytokine in the bone microenvironment, binds to its receptor, RANK, on mononuclear cell surfaces, initiating intracellular signaling cascades that facilitate cell–cell fusion and osteoclastogenesis [47]. Notably, RANKL secretion exhibited a modest yet significant increase after 14/1 d in static monoculture compared to that in the dynamic counterpart; however, no significant differences were discernable among the tested conditions after 28/14 d. Interestingly, RANKL secretion was significantly enhanced in the dynamic monoculture of hBM-MSCs after 42/28 d in culture. On the other hand, M-CSF binds to its receptor, c-Fms (colony-stimulating factor 1 receptor), on the surface of osteoclast precursors, stimulating their proliferation and survival. This process is a prerequisite for the subsequent RANKL-induced osteoclast differentiation, since together with RANKL, M-CSF synergistically enhances osteoclast formation and activation, ensuring efficient bone resorption [48]. M-CSF secretion exhibited a similar pattern as that of RANKL after 14/1 d in culture. However, after 28/14 d, M-CSF secretion was significantly diminished under dynamic compared to static conditions, a trend that persisted up to 42/28 d in culture, with a significant increase noted in the dynamic monoculture compared to the dynamic co-culture. The unaffected RANKL expression after the application of uniaxial compression confirms previous reports, suggesting that mechanical stimulation in co-culture studies selectively augments OPG production, which binds to RANKL, rather than directly influencing RANKL secretion [30].

Cell viability through F4/80 macrophage-specific marker and actin distribution staining revealed rich congregations of round-shaped cells after 7 and 14 d, of increased proliferation rate between the subsequent time points. Using TNF-α and IL-10 staining via CLSM imaging as representative M1 and M2 markers, respectively, we were able to obtain visual and quantitative cues for the phenotypic commitment of macrophages under mechanical stimulation. TNF-α promotes M1 macrophage polarization, by activating pro-inflammatory pathways, and enhances the production of inflammatory cytokines and reactive oxygen species and thus the inflammatory response. In contrast, IL-10 promotes M2 macrophage polarization through the suppression of pro-inflammatory signaling pathways and promotes tissue repair and anti-inflammatory cytokine production [15]. Uniaxial compression significantly suppressed the secreted levels of TNF-α after 7 d of culture, compared to that of the static condition, but also augmented the expression of IL-10 at the same time point. These results are consistent with findings previously reported on the cyclic mechanical loading of macrophage-laden HA-based scaffolds, with stimulation conditions clearly favoring the M2 phenotype over the M1 one [49]. Further evaluation of the immunomodulatory potential was carried out through the gene expression profiles of 2 M1 and M2 markers, iNOs and Arg1, respectively, after 7, 14, and 21 d in culture. M1 macrophages secrete the iNOs enzyme, to produce high levels of NO, while M2 macrophages produce urea and ornithine using Arg1. Therefore, the presence of iNOs or Arg1 is a hallmark of M1 or M2 polarization. Mechanical stimulation did not appear to affect iNOs expression but significantly enhanced the expression of Arg1 after 14 and 21 d in culture, showcasing the strong anti-inflammatory character that uniaxial compression imposes [49].

In summary, we succeeded in establishing an advanced dynamic BTE culture model, with strong anti-osteoporotic properties and increased anti-inflammatory attributes, negating the dependence on expensive growth factor supplementation. In total, the application of cyclic uniaxial compression imparted notable osteogenic and osteoclastogenic suppression effects, under both mono- and co-culture conditions, with concurrent enhanced directionality of macrophages toward the M2 phenotype. Combining all these key elements of bone regeneration, this study constitutes a greatly innovative approach for the development of anti-osteoporotic, 3D scaffold-based models, of amplified applicability in the field of regenerative medicine.

Herein, we present the development of a growth-factor-free co-culture, achieved via the mediation of cyclic uniaxial compression of 3D-printed Sr–nHA-containing composite scaffolds. As intrinsic immunomodulation has been found to be strongly influenced by mechanical stimulation during different bone remodeling phases, macrophage polarization was thus investigated under dynamic and static conditions. Both dynamic and static culture conditions showed adequate cell adhesion and proliferation, as determined through SEM and confocal imaging following DAPI/actin staining. The ALP and TRAP enzyme investigation, in terms of both quantification and visualization, revealed a profound effect of mechanical stimulation versus static cultures in boosting the osteogenic response of the co-culture system while concurrently significantly suppressing hPBMCs’ osteoclastogenic differentiation potential. Delving deeper into the anti-osteoporotic capacity of the proposed 3D model, the gene expression of specific markers was analyzed, showcasing enhanced up-regulation of principal osteogenic markers such as OSC, OPG, and RUNX2 and down-regulation of the osteoclastogenesis-related DC-STAMP, NFATc1, and TRAP genes, in mono- and co-cultures. ELISA detection of soluble RANKL and M-CSF growth factors from hBM-MSC culture supernatants showed comparable RANKL secretion levels for all conditions, except for BM-MSC monocultures on 42/28 d, which appeared to be highly up-regulated. Conversely, M-CSF from the dynamic co-culture illustrated a steep decline after 14/28 and 42/28 d, underlying the complementary anti-osteoclastogenic effect of the dynamic co-culture. Cyclic uniaxial compression of macrophage-seeded scaffolds did not affect cell viability and proliferation but crucially impacted the M1/M2 phenotype commitment ratio, in favor of M2 macrophages, as depicted through TNF-α and IL-10 secretion via CLSM and iNOs and Arg1 gene expression. As a next step, we plan to extend our model to animal studies to evaluate its bone regeneration potential in vivo. For instance, osteoporosis could be experimentally induced in the long bones of an animal model, followed by orthotopic implantation and stabilization of our scaffold in the bone defect. The regenerative capacity of the implant would then be assessed using micro-computed tomography imaging and histological analysis. Given that our system is designed with anti-osteoporotic properties, there is also the potential for future development to include the delivery of bone resorption inhibitor drugs, enhancing its therapeutic application in osteoporotic bone healing.

## Data Availability

All data needed to evaluate the conclusions of the study are available (such as present in the paper) upon request. There are no restrictions on data availability.
